# Kiwifruit sensitivity to boron: impact on physiological and molecular responses

**DOI:** 10.3389/fpls.2025.1549854

**Published:** 2025-03-24

**Authors:** Gudeta Chalchisa, Abu Naim Md. Muzahid, Md Dulal Ali Mollah, Edmore Gasura, Xiaodong Xie, Xiaoying Liu, Haiyan Lv, Hua Tian, Caihong Zhong, Dawei Li

**Affiliations:** ^1^ CAS Key Laboratory of Plant Germplasm Enhancement and Specialty Agriculture, Wuhan Botanical Garden, The Innovative Academy of Seed Design, Chinese Academy of Sciences, Wuhan, China; ^2^ Plant Research Department, Gullele Botanical Garden, Addis Ababa, Ethiopia; ^3^ State Key Laboratory of Plant Diversity and Specialty Crops, Chinese Academy of Sciences, Wuhan, China; ^4^ Department of Agronomy and Agricultural Extension, University of Rajshahi, Rajshahi, Bangladesh; ^5^ Department of Plant Production Sciences and Technologies, University of Zimbabwe, Harare, Zimbabwe

**Keywords:** kiwifruit, B function, boron stress, physiological response, gene regulation

## Abstract

Boron (B) is an essential micronutrient critical for crop growth and productivity. However, excessive boron concentrations can impair plant development, and detoxification remains a significant challenge. Understanding genetic variability and identifying tolerance mechanisms are crucial for developing boron-resistant cultivars. This study explores the physiological and molecular responses of two *Actinidia* species, namely kiwifruit (*A.chinensis*) and kiwiberry (*A.arguta*), to varying levels of excess B. Under excessive B conditions, B accumulation followed the order roots< stems< leaves, with maximum concentrations of 68.6 mg/kg, 105 mg/kg, and 160.7 mg/kg in *AC*, and 68.2 mg/kg, 107 mg/kg, and 196.9 mg/kg in *AA*, respectively. B toxicity symptoms appeared in *AA* when B levels exceeded 50 mg/kg, leading to a 15–20% reduction in dry weight across roots, stems, and leaves. *AC* exhibited greater sensitivity, with a 20–30% reduction in dry biomass. Both species showed significant declines in chlorophyll a and b content under B stress, with alterations in the chlorophyll a/b ratio and increased oxidative stress. Additionally, stress-responsive genes, including *1-aminocyclopropane-1-carboxylate synthase* (*Actinidia10066*) and *xyloglucan endotransglucosylase/hydrolase* (*Actinidia11948*), were downregulated in response to B stress, suggesting potential disruptions in growth and development. These findings provide valuable insights into the differential physiological and molecular responses to excess boron in *Actinidia* species, laying a foundation for functional genomics research and the development of boron-tolerant kiwifruit cultivars.

## Graphical Abstract

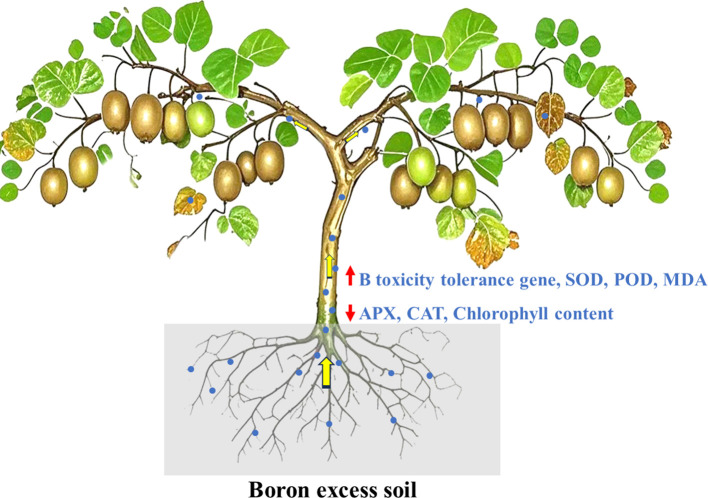

## 1 Introduction

Boron is a crucial element for the development and growth of vascular plants (Pereira et al., 2021; Bolaños et al., 2004; Shireen et al., 2018; Vera-Maldonado et al., 2024). It facilitates vital physiological processes, including carbohydrate distribution, plasma membrane transport, and cell wall stability (Chen et al., 2014; Liu et al., 2013; Shireen et al., 2018). Previous studies have established a strong correlation between optimal plant growth and adequate boron levels (Riaz et al., 2021). However, boron concentrations exceeding optimal levels can become toxic, negatively impacting plant health and growth (Miwa and Fujiwara, 2010). Different plant species exhibit varied tolerance to boron toxicity, with citrus being susceptible and some other species demonstrating higher resilience (Miwa and Fujiwara, 2010; Huang et al., 2014). Research into the morphological, physiological, and genetic responses in diverse species under excess boron stress provides critical insights for breeding programs that trigger the development of boron-tolerant varieties suitable for industrial purposes.

Excessive boron accumulation in soils presents a significant challenge, impeding growth and causing metabolic disruptions that can result in fruit deformation and progressive necrosis in leaves and stems (Reid et al., 2004; Landi et al., 2019). The primary phytotoxic effects of excess boron result from three key metabolic disturbances: (1) interference with cell division and development through binding with free ribose and RNA; (2) disruption of primary metabolism due to binding with ribose in ATP and NAD(P)H; and (3) reduction of cytosolic pH, affecting protein conformation and biosynthesis (Reid et al., 2004). The challenges in remediating boron toxicity, compounded by the limitations of current chemical and physical removal techniques, underscore the need for further research into alternative solutions (Laçin et al., 2015).

The toxic effects of excessive boron adversely affect various plant tissues, including leaves, stems, buds, fruits, and roots (Choi et al., 2007; Aquea et al., 2012; Esim et al., 2013). In leaves, boron toxicity can alter leaf thickness and disrupt the balance of photosynthetic pigments, including chlorophyll a and b, impairing photosynthetic efficiency (Zhang et al., 2023; Zou et al., 2024; Huang et al., 2014; Kayıhan et al., 2017). Reduced photosynthesis under high boron stress leads to increased reactive oxygen species (ROS) and elevated oxidative stress (Molassiotis et al., 2006; Cervilla et al., 2007; Ardıc et al., 2009; Çatav et al., 2018; Simón-Grao et al., 2019). Excess boron in stems induces the formation of cork and collenchyma cells, resulting in cell wall thickening in loquat (Papadakis et al., 2018; Wu X et al., 2019; Abdulnour et al., 2000; Wang et al., 2003), whereas it causes phloem cell wall thickening in citrus (Huang et al., 2014; Simón-Grao et al., 2018). Roots also exhibit adaptive responses, with decreased meristematic activity alongside increases in hypodermal thickening, cortical cell suberin deposition, and root lignification (Choi et al., 2007; Ghanati et al., 2002; Landi et al., 2019). These alterations indicate a detoxifying mechanism that prevents further uptake and reduces toxicity by sequestering excess boron in less sensitive tissues.

Kiwifruit (*Actinidia* spp.) is a vitamin C-rich fruit native to China, encompassing 54 species. The primary commercial varieties are *A. chinensis*, *A. deliciosa*, and *A. arguta*, which are widely cultivated in regions such as China, New Zealand, Italy, Greece, and Iran (Zhong et al., 2021). Previous studies have shown that appropriate levels of boron fertilization, such as foliar application of 0.3% sodium borate, can increase by 17.12% and dry matter content by 2.94% compared to controls (Long et al., 2015). Furthermore, boron application has been found to enhance chlorophyll content and dry matter accumulation in kiwifruit leaves, promoting photosynthesis (Ling et al., 2023). However, excessive boron fertilization can lead to B toxicity, which reduces both the yield and quality of kiwifruit. Understanding the biological role of boron, its effects, and its absorption mechanisms is thus crucial for managing boron toxicity in kiwifruit orchards (Chatzissavvidis and Antonopoulou, 2020). After boron toxicity occurs in kiwifruit, strategies such as improving irrigation practices and controlling industrial emissions has been proposed as potential methods to enhance boron tolerance in kiwifruit cultivation (Sotiropoulos et al., 2006; Zhao et al., 2024; Xia et al., 2021). Additionally, calcium application has been shown to reduce boron uptake in kiwifruit (Zhao et al., 2024; Liu et al., 2022). Numerous factors influence plant tolerance to boron toxicity, particularly those related to boron adsorption and translocation, which are crucial for improving tolerance in environments with excessive boron (Xia et al., 2021; Yıldırım and Uylaş, 2016). Research on resistance gene related to boron adsorption and translation in kiwifruit remains limited and needs systematic investigation.

This study aimed to investigate the effects of elevated boron on kiwifruit production, focusing on physiological changes, boron concentration, chlorophyll levels, and antioxidant activity. Additionally, RNA sequencing and transcriptomic approaches were utilized to assess the molecular and physiological effects of varying boron concentrations in kiwifruit. The findings provide direct evidence to explore interspecies genetic variability and facilitate breeding varieties with improved resistance to boron-excessive soils.

## 2 Materials and methods

### 2.1 Plant materials and growth conditions

This study was conducted at the National *Actinidia* Germplasm Repository, Wuhan Botanical Garden, Chinese Academy of Sciences, Wuhan, China (24°02’19” N, 109°23’48” E, 28 m elevation). Sixty kiwifruit seedlings, comprising 30 *Actinidia chinensis* cv. ‘Donghong’ and 30 *Actinidia arguta* cv. ‘Mizhan no.1’, were cultivated under varying boron (B) concentrations while maintaining consistent fertilizer and irrigation practices. Seedlings with uniform stem diameters and heights were selected. Each seedling was pruned to a small remnant to promote new stem and leaf formation in response to B treatment. Plants were grown in 6-inch plastic pots containing a 1:1 mixture of vermiculite and perlite and irrigated with Hoagland nutrient solution containing essential nutrients, including ZnSO₄·7H_2_O (8.6 mg/L), MgSO₄·7H_2_O (493 mg/L), Fe-EDTA (2.5 mL), KH_2_PO₄ (136 mg/L), K_2_HPO₄ (0.83 mg/L), KNO₃ (506 mg/L), Ca(NO₃)_2_·4H_2_O (945 mg/L), MnCl_2_·4H_2_O (22.3 mg/L), (NH₄)₆Mo₇O_2_₄ (80 mg/L), and CuSO₄·.5H_2_O (0.025 mg/L) (Hoagland and Arnon, 1950). All chemicals used were high purity, and deionized water was used for irrigation. The pH of the solution was maintained at 6.0 by adding 2 mL ultrapure water or NaOH every two weeks. Watering was carried out every three days throughout the experiment. Five different boron concentrations were applied: WT (0 mg/L), 0.6 mg/L, 3 mg/L, 6 mg/L, and 9 mg/L B, with boron derived from H₃BO₃. The 0 mg/L B concentration was considered the wild type (WT), while 0.6 and 3 mg/L B were deemed adequate, and 6 and 9 mg/L B represented excess levels. Each treatment was replicated six times per genotype, resulting in 60 seedlings.

### 2.2 Experimental design and boron determination

Five boron treatments were applied to each of the two kiwifruit species in a completely randomized design with six replications. Boron treatments started on July 1, with the five B concentrations (WT, 0.6, 3, 6, and 9 mg/L B) applied over a week (Abdulnour et al., 2000). Leaf samples were collected from each genotype exhibiting toxicity symptoms to determine the B concentrations. Samples were oven-dried at 68°C for three days, then digested with 5 mL concentrated nitric acid and 1 mL hydrogen peroxide in a microwave system (ETHOS ONE, Milestone, Italy). The digested solution was neutralized, diluted to a final volume of 50 mL, and analyzed using inductively coupled plasma optical emission spectroscopy (ICP-OES, Optima 2000DV, Perkin-Elmer, USA) with a recovery rate of over 94% verified using control standards (GBW07604).

### 2.3 Plant growth parameter and symptoms analysis

At the end of the experiment stem length, stem diameter, leaf length, and leaf width of each seedling were measured using a digital Vernier caliper (DL 91150) and a 30 cm steel ruler. Stem and root dry weights were recorded after the samples were dried. Leaf samples from each boron (B) treatment were prepared for scanning electron microscopy (SEM) analysis. This process involved drying the samples with a carbon dioxide critical point dryer (CPD300, Leica), coating them with gold using a sputter coater (MC1000, Hitachi), and imaging with a scanning electron microscope (TM3030, Hitachi) (Sotiropoulos et al., 2002).

### 2.4 Photosynthesis and chlorophyll content measurement

Photosynthesis was measured using the SPAD-502 chlorophyll meter (Konica Minolta, Japan) after differences between wild-type (WT) and B-toxicity treatments were observed. Chlorophyll content was determined from a 0.20 g fresh leaf sample prepared according to standard protocols (Hiscox and Israelstam, 1979). Samples were incubated with dimethyl sulfoxide in an 8 mL centrifuge tube at 28°C in a dark environment with 200 rpm agitation for 72 hours. Absorbances at 470 nm, 645 nm, and 663 nm were recorded with an ultraviolet spectrometer, and chlorophyll a, chlorophyll b, total chlorophyll, and the chlorophyll a/b ratio were calculated as follows:


$$\text{Chll a}=\frac{\left(12.7\;\times \text{ OD}663-2.69\;\times \text{ OD}645\right)\;\times \text{ VT }}{\text{W }\times \;1000\;\times \text{ VS}}$$

$$\text{Chll b}=\frac{\left(22.9\;\times \text{ OD}645-4.68\;\times \text{ OD}663\right)\;\times \text{ VT}}{\text{W }\times \;1000\;\times \text{ VS}}$$

Chll total=chll a +chll b

$$\text{Chll}\left(\text{a}/\text{b}\right)=\frac{\text{chll a}}{\text{chll b}}$$

Where VT is the total volume (mL) of the extract, VS is the volume of the mixed-in cuvette (µL), and W is the sample fresh weight (g).

### 2.5 Determination of antioxidant enzyme activities and contents of malondialdehyde (MDA)

To analyze superoxide dismutase (SOD) and catalase (CAT) activity, freeze 0.1 g of leaf segments in liquid nitrogen. Mix the frozen pieces with 2 mL of ice-cold phosphate buffer (1 M KH2PO4, pH 6.7), 0.1 g of polyvinylpyrrolidone (PVP), and 20 µL of phenylmethylsulfonyl fluoride (PMSF, 1 mM). Blend the mixture and centrifuge it at 12000 g at 4°C for 10 minutes. Use the supernatant to measure SOD activity at 560 nm and CAT activity at 240 nm (Yan et al., 2021). A 0.1 g sample of fresh tissue was homogenized with 10% trichloroacetic acid (TCA) and centrifuged at 12000 g for 10 minutes. Then, 200 µL of the extract was combined with an equal volume of 20.0% (w/v) TCA and a solution containing 20.0% TCA plus 0.65% thiobarbituric acid (TBA). The mixture was heated at 95°C for 30 minutes, cooled in an ice bath, and centrifuged again at 12000 g for 10 minutes. Malondialdehyde (MDA) content was determined at 532 nm and 600 nm (Imran et al., 2020). Additionally, activities of ascorbate peroxidase (APX) at 290 nm and peroxidase (POD) at 470 nm were quantified using assay kits (Beijing Solarbio Science & Technology Co., Ltd.).

### 2.6 RNA extraction, qPCR analysis, RNA-seq analysis

Total RNA was extracted from *A. arguta* leaves using the RePure Plant RNA Kit (Magen, Guangzhou, China). Following quality verification, cDNA synthesis was performed with the One-Step gDNA Removal and cDNA Synthesis SuperMix kit (TransGen, Beijing, China). RNA-seq libraries were prepared, and Illumina sequencing was conducted for wild-type (WT, 0 mg/L) and boron treatments (3 mg/L and 9 mg/L) in *A. arguta*. The RNA-seq reads were processed using Trimmomatic to remove low-quality sequences, and clean reads were aligned to the kiwifruit v3 genome reference (Wu H, et al., 2019). Differentially expressed genes (DEGs) were identified using DESeq2 (version 1.26.0) with thresholds of *P*-value< 0.05 and |log2FoldChange| ≥ 2 (Love et al., 2014). Gene Ontology (GO) (Ashburner et al., 2000) and KEGG Pathway (Kanehisa et al., 2004) enrichment analyses were performed using hypergeometric tests. Enrichment results were analyzed and visualized with the cluster Profiler software (version 3.14.3) in R (v4.0.2) (Yu et al., 2012). Heatmaps were generated using TBtools (Chen et al., 2020). Protein-protein interaction networks for boron-responsive genes were constructed and visualized in Cytoscape (v3.10.2). Quantitative PCR (qPCR) was conducted using the TransStart^®^ Green qPCR SuperMix kit (TransGen) as described by Wang et al. (2018), with three biological replicates per treatment. Gene expression validation was performed using the ΔΔCt method, and primer sequences are provided in **Supplementary Table 2**. The RNA-seq data have been deposited in GenBank (NCBI) under accession number PRJNA1144175.

### 2.7 Statistical analysis

Statistical analyses were conducted using a completely randomized design, comprising five treatments with 60 plants, each was replicated six times. Gene Ontology (GO), Kyoto Encyclopedia of Genes and Genomes (KEGG), Volcano plots, Venn diagrams, and metabolic pathway data were executed using R software (version 4.4.0). The cluster heatmap was generated with TBtools (version 10.4.0), while protein-protein interaction networks were constructed using Cytoscape (version 3.10.2). Graphical representations were generated with GraphPad Prism (version 10.3.0), and tabular data were organized in Excel 2019. The statistical significance of observed effects was assessed using analysis of variance (ANOVA), with *post-hoc* comparisons performed utilizing t-tests. A significance threshold of *P*< 0.05 was applied (IBM SPSS Statistics 23) to evaluate differences between treatments and genotypes.

## 3 Results

### 3.1 Exogenously applied boron increased internal boron concentration in different tissues

**Figure 1:**
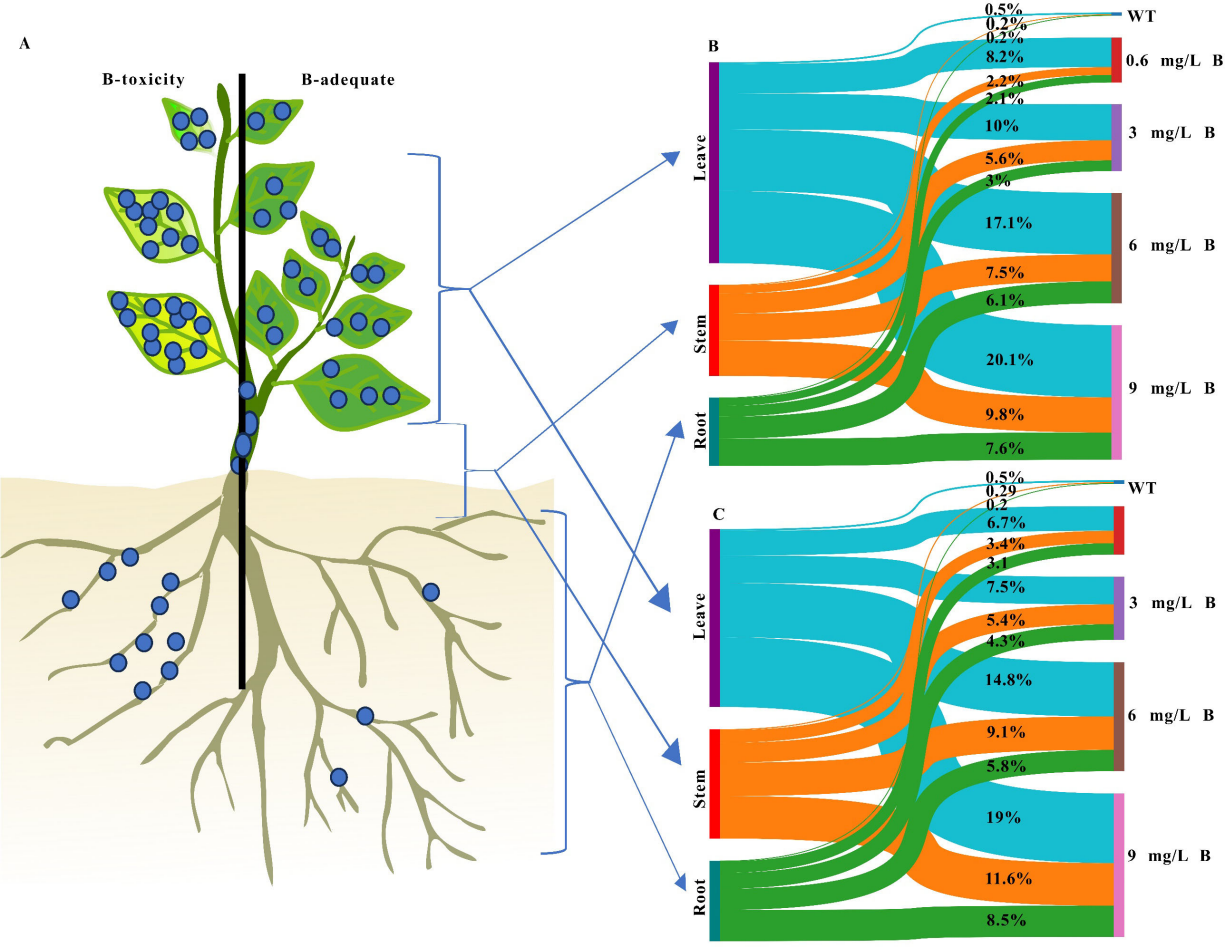
The proportion of B content in the leaf, stem, and root of **(A)** kiwifruit tree, **(B)** *AA*, and **(C)** *AC* kiwifruits were measured under five levels of B content. The line width indicates the proportion of stocks, and the box height indicates the percentage of total B content in each tissue.

Exogenous boron (B) application in kiwifruit species resulted in differential B accumulation across various tissues. Before treatment, soil B levels were deficient (0.070 mg/kg), and initial B concentrations in roots, stems, and older leaves were 0.26, 0.27, and 0.30 mg/kg, respectively. As B treatment levels increased, the most pronounced accumulation occurred between 3 mg/L and 6 mg/L, with B content in *A. arguta* leaves increasing 92-fold within this range. Under a 9 mg/L B treatment, B concentrations in *AA* tissues peaked at 78.2, 101, and 196.9 mg/kg in roots, stems, and older leaves, respectively (**Figure 1B**), representing increases of 78, 80, and 95 times compared to the wild type. Significant differences were also observed between the two kiwifruit species, *A. chinensis* and *A. arguta*. Under the same 9 mg/L treatment, B concentrations in *AC* tissues reached 70–98% of those in *AA*, with values of 73.4, 98.4, and 160.7 mg/kg in roots, stems, and older leaves, respectively (**Figure 1C**).

**Figure 1** illustrates the distribution of boron (B) in the leaves, stems, and roots of *A. arguta* and *A. chinensis* kiwifruit plants across five B treatment concentrations. The Sankey diagram highlights the leaves with the highest B levels under B stress. *AA* and *AC* leaves retained 55.86% and 48.48% of the total B, respectively. In contrast, B concentrations in *AC* stems and roots were 29.71% and 21.81%, while *AA* stems and roots contained lower proportions, at 25.21% and 18.94%, respectively. B translocation efficiency was assessed using transport factors (TFs) for root-to-stem (TF stem/root) and root-to-leaf (TF leaf/root) transport. *AA* exhibited a stronger TF leaf/root than *AC*, indicating a higher capacity for boron translocation to the leaves. However, *AC* demonstrated a greater TF stem/root (**Supplementary Table 3**). These findings suggest that both kiwifruit species, *A. arguta* and *A. chinensis*, are highly vulnerable to boron toxicity in their young organs. Conversely, the efficient leaf-to-root translocation in kiwifruit facilitates effective boron uptake from the soil, potentially aiding in the mitigation of soil contamination.

### 3.2 Plant growth and symptoms under B stress

**Figure 2:**
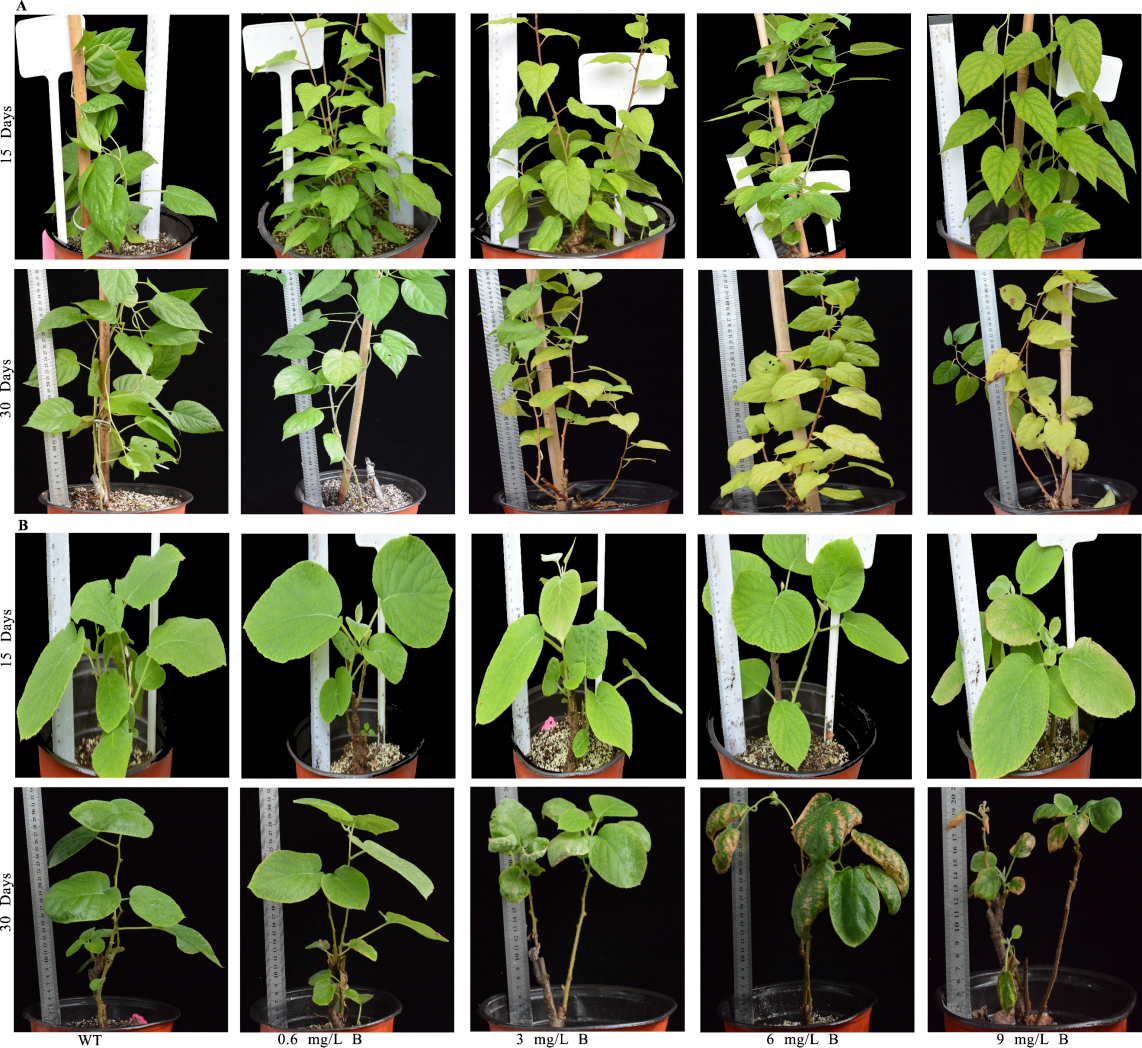
The boron toxicity symptoms exhibited by *Actinidia. arguta* **(A)** and *A*. *chinensis* **(B)** plants under different boron concentrations after 15 and 30 days of treatment.

**Figure 3:**
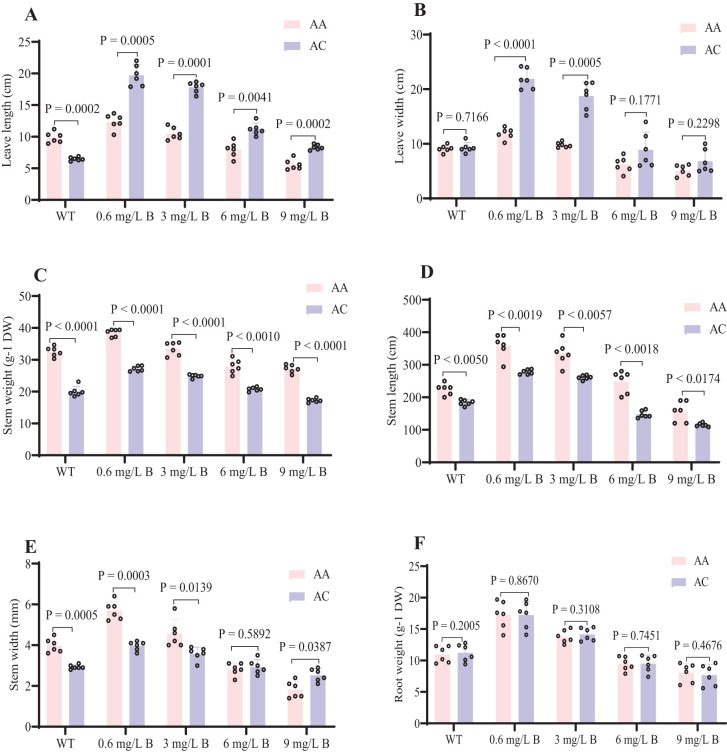
Kiwifruit seedling physiological parameters at different B concentrations. **(A)** Leaf length (cm) **(B)** Leave width (cm) **(C)** Stem weight (g-1 DW) **(D)** Stem length (cm) **(E)** Stem width (mm) **(F)** Root weight (g-1 DW). The Kiwifruit seedlings were treated with WT, 0.6, 3, 6, and 9 mg/L of B concentration of AC and AA. The significant difference among treatments by t-tests at P < 0.05.

Boron toxicity significantly inhibited kiwifruit growth and development, with notable differences in growth parameters and dry matter accumulation across various B concentrations (**Figures 2A, B**). Symptoms of B toxicity, such as leaf chlorosis and growth inhibition, appeared within two weeks of treatment. Physiological parameters were significantly altered by B exposure over one month. At 3 mg/L B, growth was notably suppressed, with delayed leaf emergence and a color change in leaves from green to dark brown (**Figures 2A, B**). At 6 mg/L B, *AC* exhibited reductions in several growth parameters, including stem height (1.47 m), stem width (2.9 cm), stem dry weight (20.7 g), leaf length (13.5 cm), leaf width (8.9 cm), and root dry weight (9.5 g), showing declines of 20-50% compared to WT (**Figure 3**). Similarly, *AA* exhibited reductions in growth parameters (e.g., stem height, leaf length), but these were less pronounced (15-48%) than in *AC*. *AA* showed more excellent resistance to B toxicity than *AC*, as reflected by less overall growth inhibition.

**Figure 4:**
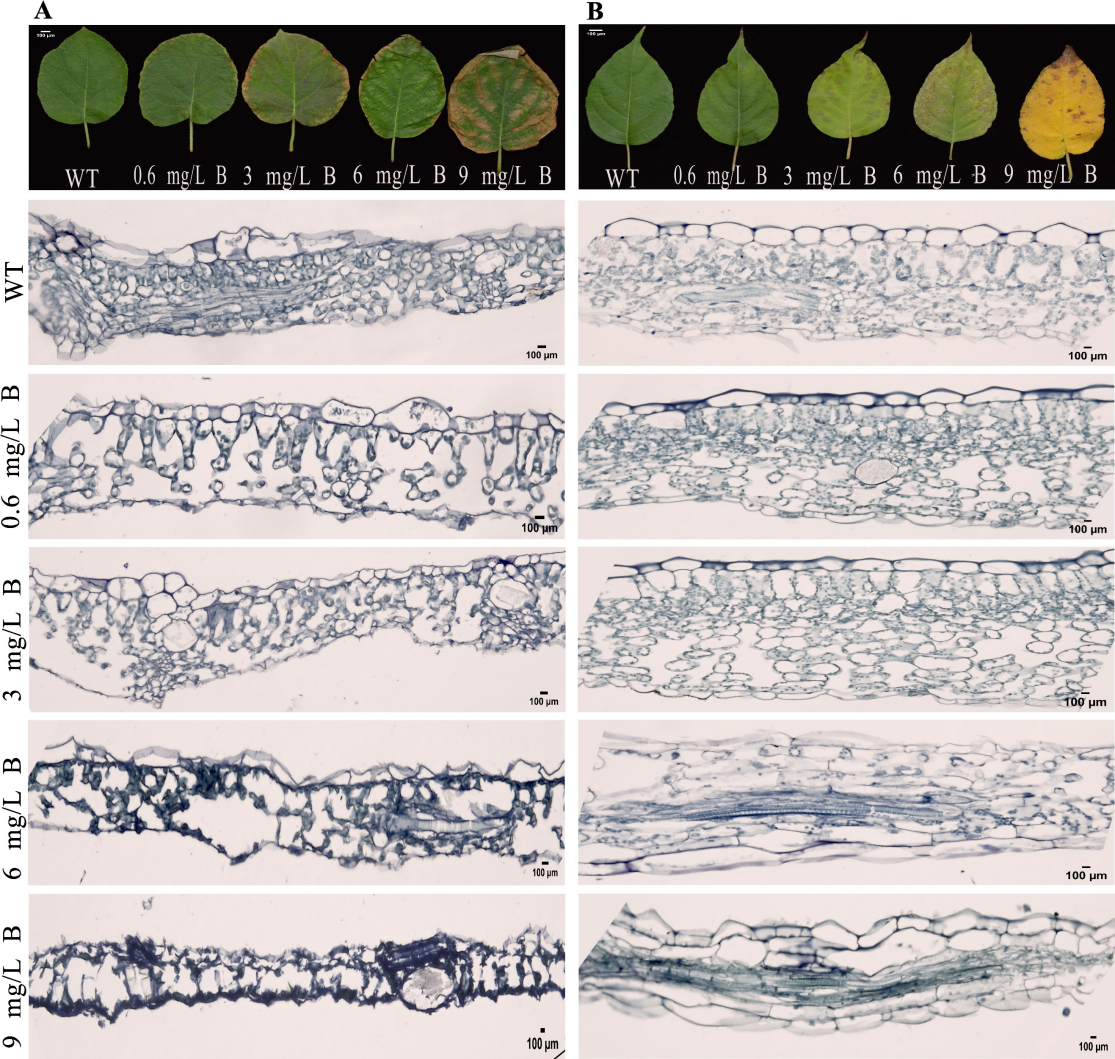
Micrographs of Cell Morphology in *A*. *chinensis* **(A)** and *A*. *arguta* **(B)** Leaves Exposed to Five Boron Concentrations (WT, 0.6, 3, 6, and 9 mg/L): Visualizing Upper Epidermis (UE), Palisade Cells (PC), and Lower Epidermis (LE), Scale Bar = 100 µm.

Microscopic analysis of kiwifruit leaves under B stress revealed structural changes. Both wild-type *AC* and *AA* leaves showed typical epidermal cell sizes and well-organized palisade and spongy mesophyll cells. However, *AC* leaves treated with 0.6 mg/L and 3 mg/L B for 15 days exhibited a reduction in palisade and spongy mesophyll cell density and size (**Figure 4A**). In contrast, *AA* leaves maintained normal epidermal cell sizes, with only a slight reduction in mesophyll density, indicating milder toxicity symptoms (**Figure 4B**). At B concentrations exceeding 6 mg/L, both *AC* and *AA* leaves exhibited severe toxicity, including marked shrinkage and necrosis of cellular tissues, with *AC* leaves being more sensitive to boron toxicity at lower concentrations.

### 3.3 Photosynthesis and chlorophyll concentration

**Figure 5:**
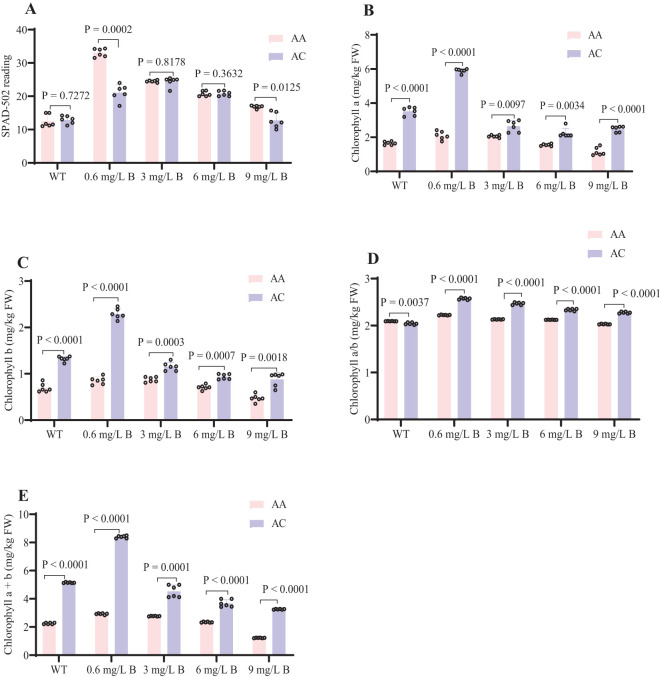
Effects of Boron on SPAD Readings and Chlorophyll a and b Concentrations in AA and AC under Different Treatments. **(A)** SPAD-502 reading **(B)** Chlorophyll a (mg/kg FW) **(C)** Chlorophyll b (mg/kg FW) **(D)** Chlorophyll a/b (mg/kg FW) **(E)** Chlorophyll a+b (mg/kg FW). Error bars represent ± SD from six biological replicates (t-test, P < 0.05). WT (0), 0.6, and 3 mg/L represent optimal B levels; 6 and 9 mg/L represent excess B levels.

Excessive B treatments negatively impacted the chlorophyll content in kiwifruit leaves. Under prolonged irrigation, the chlorophyll concentrations in the *AA* and *AC* leaves remained low, with SPAD values of 16 and 13, respectively. The highest levels of chlorophyll a and b were observed with treatments of 0.6 and 3 mg/L B, which corresponded to SPAD values of 34 and 24 in the *AA* leaves. As B concentrations increased, chlorophyll a, b, and total chlorophyll levels declined progressively. The chlorophyll a/b ratio remained stable, indicating that both chlorophyll a and b were equally affected by boron toxicity (**Figure 5**). *AC* leaves exhibited higher overall chlorophyll concentrations compared to *AA*, likely due to species-specific traits. Both kiwifruit genotypes demonstrated tolerance to boron stress when subjected to treatment with 0.6 mg/L and 3 mg/L of boron.

### 3.4 Effects of B stress on antioxidant activity and MDA levels

**Figure 6:**
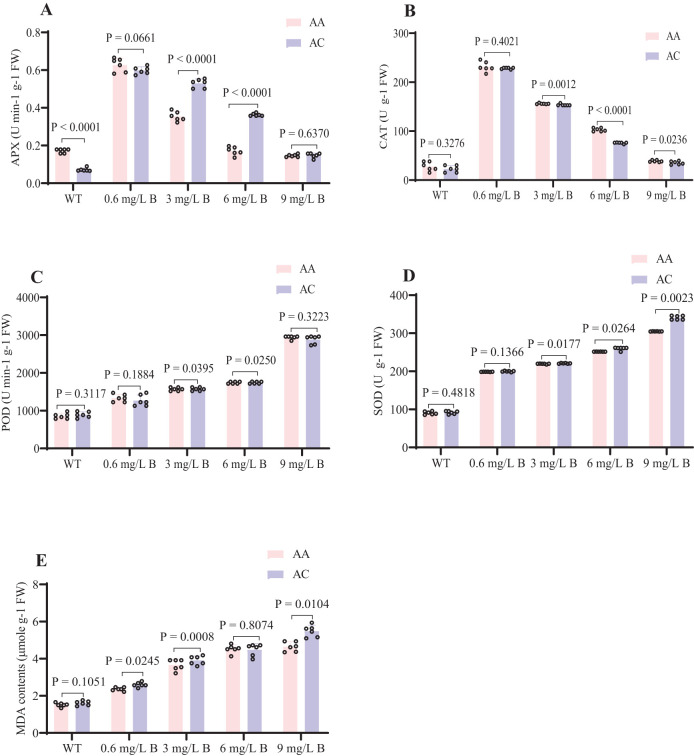
Effects of B concentration on the antioxidant system of kiwifruits. **(A)** APX (U min-1 g-1 FW) **(B)** CAT (U g-1 FW) **(C)** POD (U min-1 g-1 FW) **(D)** SOD (U g-1 FW) **(E)** MDA (µmole g-1 FW). Kiwifruits were treated with WT, 0.6, 3, 6, and 9 mg/L B concentrations in AA and AC species. The error bar represents ± SD of six biological replicates at t-test P < 0.05. WT (0), 0.6 and 3 mg/L B as efficient B content, 6 and 9 mg/L B as excess B content.

The antioxidant defense systems of the two kiwifruit species (*AC* and *AA*) exhibited similar physiological responses under boron stress. Notably, APX and CAT activities in both species sharply declined as boron concentration increased from 0.6 to 9 mg/L, with APX decreasing by 10.4% in *AC* and 2.9% in *AA*, and CAT decreasing by 7.2% in *AC* and 7.1% in *AA* at 9 mg/L B (**Figure 6**). Conversely, boron stress led to increased levels of MDA, POD, and SOD in both species (Tombuloglu et al., 2015). SOD activity rose by 28.66% in *AC* and 28.6% in *AA*, MDA levels increased by 28.7% in *AC* and 29.1% in *AA*, and POD levels increased by 31.7% in *AC* and 28.9% in *AA* under boron stress (**Figure 6**). These results suggest that *AA* is better adapted to boron stress than *AC*. The application of boron at concentrations of 0.6 mg/L and 3 mg/L significantly enhances antioxidant activity and increases tolerance to boron stress in both *AC* and *AA*.

### 3.5 The boron-related gene expression profile

**Figure 7:**
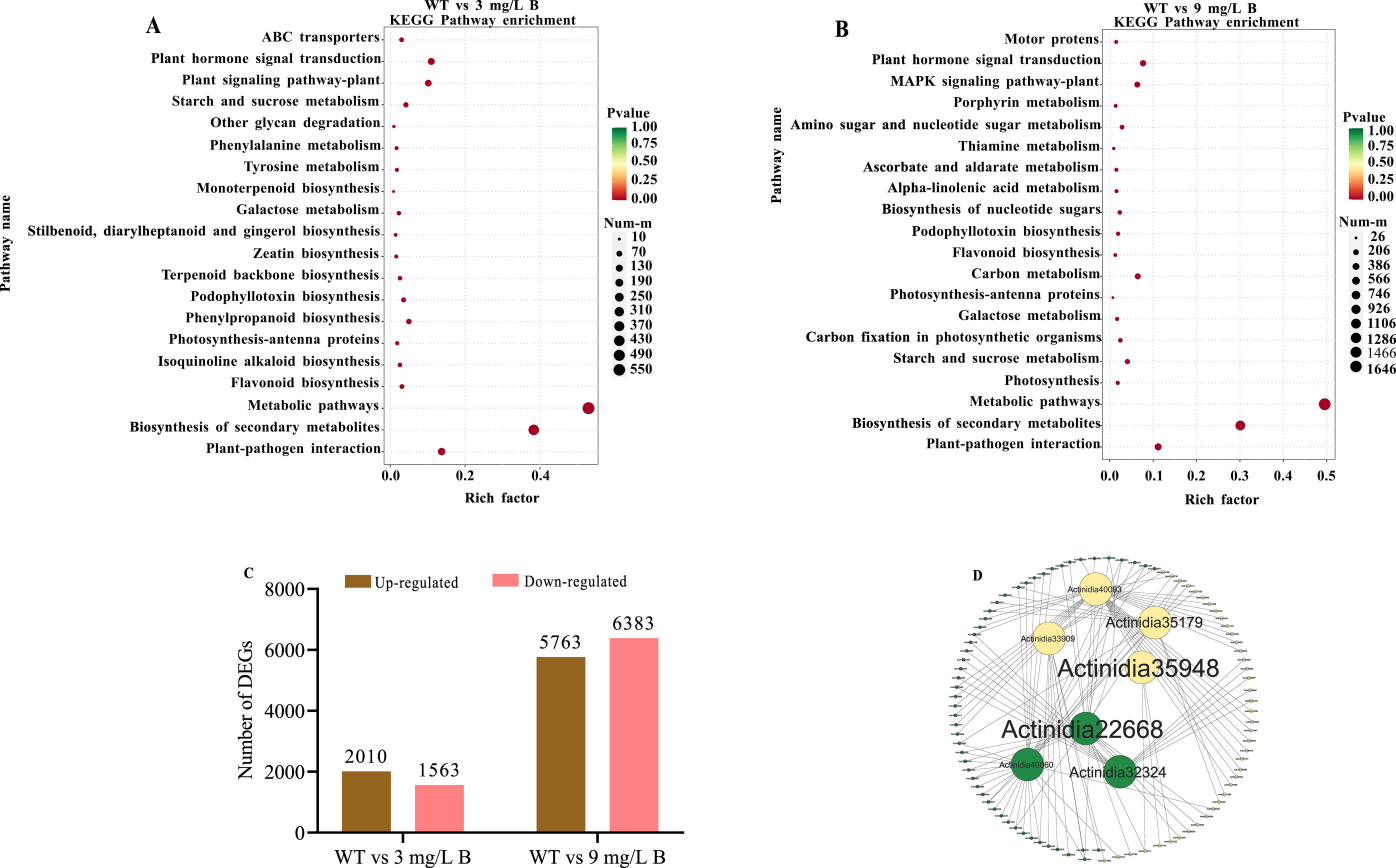
Analysis of differentially expressed genes (DEGs) related to boron **(B)** stress in *A*. *arguta* includes **(A)** KEGG enrichment of DEGs of WT vs 3 mg/L B, and **(B)** WT vs 9 mg/L B, **(C)** intraspecific pairwise comparison of kiwifruit treated with different B concentrations **(D)** Protein-protein interactions in kiwifruit genes affected by boron, with red for upregulated and yellow for downregulated genes. .

Following boron concentration assessments in wild-type (WT) and boron-treated *A. arguta* leaves (**Figure 1**), RNA-seq analysis was conducted to identify gene expression changes under boron stress. After removing adaptor sequences and low-quality reads, WT, 3 mg/L B, and 9 mg/L B treatments generated 43,318,730, 42,063,369, and 43,335,754 clean reads, respectively. The kiwifruit reference transcriptome showed over 98% Q20 scores, over 95% Q30 scores, and a GC content exceeding 44%, indicating a high-quality sequencing library suitable for accurate gene expression analysis (**Supplementary Table 1**). Transcriptomic analysis revealed a clear separation between boron treatments and WT and boron-treated samples. Two critical comparisons were made: WT vs 3 mg/L B and WT vs 9 mg/L B. In the WT vs 3 mg/L B group, 3,573 differentially expressed genes (DEGs) were identified, with 2,010 upregulated and 1,563 downregulated genes. In WT vs 9 mg/L B, 12,146 DEGs were observed, with 5,763 upregulated and 6,383 downregulated genes (**Figure 7C**). Notably, the majority of DEGs were downregulated in the WT vs 9 mg/L B comparison, indicating a solid response to high boron stress. Venn diagram analysis showed that the highest number of DEGs (36,256) was found in the WT vs 3 mg/L B comparison, while the lowest number (35,682) was recorded in WT vs 9 mg/L B (**Supplementary Figure 1F**).

The KEGG pathway analysis identified 1,834 and 4,955 differentially expressed genes (DEGs) across 20 pathways for WT compared to 3 mg/L B and 9 mg/L B, respectively, metabolic pathways were the most enriched in both comparisons (**Figures 7A, B**). The heatmap showed gene expression changes in WT vs 3 mg/L B and WT vs 9 mg/L B. In the first comparison, Rhamnogalacturonate lyase (*Actinidia06502*) and AOP3 (*Actinidia28035*) were upregulated, while Cytochrome P450 (*Actinidia12073*) and Expansin (*Actinidia38761*) were downregulated. In the second comparison, *Actinidia06502* and *Actinidia28004* increased, whereas Xyloglucan endotransglucosylase (*Actinidia11948*) and Hexosyltransferase (*Actinidia01673*) decreased (**Supplementary Figure 3A**).

The Gene Ontology (GO) enrichment analysis of differentially expressed genes (DEGs) identified 62,032 annotated DEGs. Of these, 30,971 genes (49.93%) were associated with the comparison between wild type (WT) and 3 mg/L boron (B), while 31,061 genes (50.07%) were linked to the comparison between WT and 9 mg/L B (**Supplementary Figures 1A, B**). Key findings included prevalent Biological Process terms such as the regulation of transcription and translation. Significant Cellular Component terms highlighted integral membrane components and the nucleus, while important Molecular Functions included ATP and metal ion binding. Principal Component Analysis (PCA) was performed on the reliability among WT vs 3 mg/L B and WT vs 9 mg/L B DEGs (**Supplementary Figure 3B**). The volcano plot showed that in the WT vs 3 mg/L B comparison, there were 515 upregulated and 314 downregulated genes, while in the WT vs 9 mg/L B comparison, there were 1,522 upregulated and 486 downregulated genes (**Supplementary Figures 1C, D**). Lastly, the analysis of transcription factors (TFs) identified 21 families, with WRKY and NAC being upregulated and MYB and bHLH being downregulated (**Supplementary Figure 1E**).

### 3.6 Boron-affected genes related to metabolic function and protein-protein network interaction

The metabolic function of boron was investigated by examining boron-related pathways at WT vs 3 mg/L B and 9 mg/L B (**Supplementary Figure 2**). Several DEGs associated with cysteine/methionine metabolism, phenylpropanoid biosynthesis, inositol phosphate metabolism, and the MAPK signaling pathway were increased, as well as some genes related to protein processing in the endoplasmic reticulum under boron stress. On the other hand, DEGs involved in inositol phosphate metabolism, starch, and sucrose metabolism, plant hormone signal transduction, genetic information processing, and the MAPK signaling pathway were decreased by boron stress. Specifically, several DEGs involved in the MAPK signaling pathway were upregulated, such as the Pathogenesis-related protein 1 gene (*Actinidia28004*) and the Ethylene-responsive transcription factor 1 B-like gene (*Actinidia40060).* At the same time, most of the transcription factors MYC2 (i.e., *Actinidia15025 and Actinidia28094*) and FLS2 LRR receptor (i.e., *Actinidia36397 and Actinidia37808*) were downregulated by boron stress; this pathway contributes to boron tolerance. Additionally, plant hormone signal transduction genes (i.e., *Actinidia11948, Actinidia37884*), inositol phosphate metabolism genes (i.e., *Actinidia11585* and *Actinidia23530*), and starch and sucrose metabolism genes (i.e*., Actinidia19470, Actinidia28869*) were also downregulated under boron stress (**Supplementary Figure 2**).

The protein-protein interaction (PPI) network analysis revealed 99 nodes and 113 edges. Significant differences in gene expression levels were observed. Notably, pectate lyase (*Actinidia35948*), *Actinidia35179* (9-cis-epoxy carotenoid dioxygenase), *Actinidia33909* (ferritin), and *Actinidia40093* (Auxin-responsive protein) were identified as highly downregulated genes (**Figure 7D**). The auxin-responsive protein (*Actinidia40093*) demonstrated significant interactions with downregulated genes, supporting earlier research on auxin’s role in plant stress responses. Our findings indicate that genes related to boron stress and auxin interactions were significantly downregulated (**Figure 7D**).

## 4 Discussion

Boron (B) is indispensable for plant growth and development, serving critical roles in cytoplasmic synthesis and cell wall formation by cross-linking rhamnogalacturonan II, a key cell wall component (Hu and Brown, 1994; O’Neill et al., 2001). However, excessive B uptake, particularly under conditions of high transpiration, disrupts osmotic balance, nutrient absorption, and the redistribution of essential metabolites such as starch and sugars (Macho-Rivero et al., 2018; Reid et al., 2004). This dual role of B as both an essential micronutrient and a stressor underscores its importance in agricultural systems. High B levels have been shown to significantly inhibit plant growth, as evidenced in rice and citrus rootstocks (de Abreu Neto et al., 2017; Wu et al., 2019). Our findings highlight that kiwifruit leaves accumulated the highest B concentrations among all treatments, consistent with the role of transpiration streams in B transport (Rees et al., 2011). Boron’s movement through roots and vascular tissues is critical for growth, but its excessive accumulation can impair growth, particularly in stems. Resistance mechanisms, such as efflux systems, help mitigate B toxicity. Similar trends have been reported in barley and wheat, where B toxicity resistance is associated with lower tissue B ratios (Sutton et al., 2007; Paull et al., 1991). Notably, low B treatments (0.6 and 3 mg/L B) improved growth in kiwifruit compared to the wild type (WT) and plants subjected to toxic conditions, aligning with findings in other crops (Sotiropoulos et al., 2002).

Excessive B induces physiological and biochemical disruptions, often accompanied by oxidative damage (Camacho-Cristóbal et al., 2015; Han et al., 2008; Kayıhan et al., 2016; Oiwa et al., 2013). Morphological changes, such as chlorosis and necrosis, were observed in kiwifruit leaves under B stress, particularly in the epidermal layers, leading to chlorosis at leaf tips that extended along the margins and culminated in significant leaf drop (Melnyk et al., 2005; Arredondo and Bonomelli, 2023). Root and stem development were also inhibited under high B conditions, manifesting as chlorosis and necrosis (Wimmer et al., 2015; Arredondo and Bonomelli, 2023). The observed toxicity symptoms in kiwifruit aligned with these phenotypes, confirming its sensitivity to excessive B. In addition, antioxidant enzyme activity and lipid peroxidation serve as indicators of plant sensitivity to B toxicity. Enhanced antioxidant activity under B stress has been documented in multiple crops (Zhou et al., 2017; Moustafa-Farag et al., 2016). In our study, *Actinidia arguta* (*AA*) demonstrated a more robust antioxidant response compared to *Actinidia chinensis* (*AC*). Wild-type (WT) plants exhibited reductions in superoxide dismutase (SOD), malondialdehyde (MDA), and catalase (CAT), whereas B toxicity led to increased MDA, peroxidase (POD), and SOD in both species, indicating the greater resilience of *A. arguta* (Riaz et al., 2021; Shah et al., 2017; Tombuloglu et al., 2015).

Transcriptomic analysis revealed that boron stress significantly altered gene expression in kiwifruit, highlighting pathways and genes associated with boron metabolism and stress responses. Under excessive B conditions, metabolic pathways related to cysteine, methionine, and phenylpropanoid biosynthesis were downregulated, while inositol phosphate metabolism was significantly activated (**Supplementary Figure 2**). Elevated expression of inositol oxidase 1 (*Actinidia15291*), involved in ascorbic acid synthesis, supports its role in enhancing antioxidant production under B stress (Michailidis et al., 2023; Lukaszewski and Blevins, 1996). Conversely, reduced expression of cinnamyl alcohol dehydrogenase (*Actinidia35356*), a key enzyme in lignin biosynthesis, indicated that B stress affects cell wall rigidity (Michailidis et al., 2023). Auxin-responsive genes, such as *Actinidia40093*, were implicated in growth adaptation under B stress. Auxins are critical for cellular elongation, division, and differentiation. Studies have shown that specific auxin response factors (ARFs) regulate distinct developmental processes. Our findings suggest that *Actinidia40093* plays a pivotal role in mediating growth inhibition under B stress, underscoring its importance in plant adaptation to varying boron levels.

Identifying B-resistant genes or those encoding boric acid transporters, such as *BOR1* and *NIP5*, is crucial for managing B toxicity (Takano et al., 2002, 2006). Previous research has demonstrated that *Arabidopsis* MYB transcription factors AtMYB13 and AtMYB69 enhance B tolerance without altering B levels (Nozawa et al., 2006). Similarly, suppressing the NAC-like factor BET-1 improved B tolerance in rice (Ochiai et al., 2011). Transcription factors such as WRKY42 and MYB44 regulate genes associated with B transport, ROS scavenging, and cellular homeostasis, playing vital roles in B-stress responses (Feng et al., 2021; Singhal et al., 2023; Wang et al., 2018).

Our transcriptomic analysis identified WRKY41 and NAC33 homologs in kiwifruit as potential regulators of B toxicity tolerance (**Supplementary Figure 4**). These findings, combined with the differential expression of metabolic genes such as *Actinidia15025*, *Actinidia36397*, and *Actinidia11948*, highlight the intricate regulatory networks governing B stress responses. Protein-protein interaction analyses further revealed *Actinidia33909* and *Actinidia40093* as central nodes, emphasizing their functional relevance in B toxicity tolerance.

Several strategies have been employed to mitigate boron toxicity in crops, including agronomic, chemical, and biological approaches. Effective irrigation and soil management practices, such as improving drainage and using low-boron irrigation water, help leach excess borron from the rhizosphere (Sotiropoulos et al., 2006). Adjusting soil pH can optimize boron availability (Zhao et al., 2024), while calcium amendments, such as calcium carbonate or sulfate, reduce boron uptake by altering soil ionic balance (Zhao et al., 2024; Liu et al., 2022). However, while these short-term interventions provide immediate relief, long-term solutions must focus on breeding boron-resistant crop varieties. Breeding such varieties is critical for enhancing agricultural productivity and ensuring the sustainability of industries reliant on boron-sensitive crops, such as kiwifruit, citrus, and grains. Advances in molecular genetics have identified genotypes with inherent tolerance to boron toxicity, particularly those exhibiting reduced boron uptake and efficient vacuolar sequestration (Princi et al., 2016; Hua et al., 2021). Our research has also identified variations in boron tolerance among different kiwifruit genotypes and uncovered boron toxicity response genes, laying the groundwork for biotechnological applications in breeding resistant varieties. In the future, molecular tools like Marker-Assisted Selection (MAS) will facilitate the identification of boron tolerance-related genotype and genes (Emebiri et al., 2009), supporting the development of resistant varieties.

## 5 Conclusion

This study demonstrates that excess boron (B) significantly impacts key physiological parameters in kiwifruit, including chlorophyll concentration, Boron accumulation, and antioxidant activity. The *Actinidia chinensis* genotype shows a higher sensitivity to boron (B) stress compared to *Actinidia arguta*, emphasizing the genotype-specific responses identified in this study. This research clarifies the physiological and molecular effects of boron excess in kiwifruit, highlighting the importance of dose-dependent reactions. Key transcription factors, such as WRKY41 and NAC33, along with stress-responsive genes like *Actinidia17814* and *Actinidia24652*, play crucial roles in responding to boron (B) toxicity. Additionally, metabolic genes (e.g., *Actinidia15025*, *Actinidia36397, Actinidia11948*) and protein-protein interaction hubs (*Actinidia33909* and *Actinidia40093)* highlight the complex regulatory networks that govern adaptation to B stress. These findings provide critical insights into the transcriptional and metabolic reprogramming triggered by B toxicity. They establish a foundation for future research to unravel specific regulatory mechanisms and interactions, ultimately enabling the development of B-tolerant kiwifruit cultivars. Such advancements will support sustainable crop production in high-B environments.

## Data Availability

The datasets presented in this study can be found in online repositories. The names of the repository/repositories and accession number(s) can be found in the article/**Supplementary Material**.
