# Supplementary material for: Kiwifruit sensitivity to boron: impact on physiological and molecular responses
Source: Front Plant Sci. 2025 Mar 24;16:1549854. doi: 10.3389/fpls.2025.1549854 (PMC11973261; doi:10.3389/fpls.2025.1549854)
Supplement: Supplementary file 1 [file DataSheet1.docx]

**Supplementary Table 1. Statistics on the amount of sequencing data related to B stress genes**

| Sample | Raw reads | Raw bases | Clean reads | Clean bases | Q20_rate | Q30_rate | GC_content |
| --- | --- | --- | --- | --- | --- | --- | --- |
| 3 mg/L B-1 | 39,593,896 | 5,939,084,400 | 39,549,630 | 5,737,656,356 | 98.26% | 95.21% | 44.79% |
| 3 mg/L B-2 | 43,340,624 | 6,501,093,600 | 43,293,260 | 6,236,856,027 | 98.18% | 94.98% | 44.49% |
| 3 mg/L B-3 | 43,396,298 | 6,509,444,700 | 43,347,218 | 6,301,482,402 | 98.48% | 95.66% | 44.83% |
| 9 mg/L B-1 | 43,349,820 | 6,502,473,000 | 43,323,718 | 6,172,234,897 | 98.17% | 94.90% | 45.02% |
| 9 mg/L B-2 | 43,365,710 | 6,504,856,500 | 43,340,140 | 6,207,405,711 | 98.20% | 95.02% | 45.03% |
| 9 mg/L B-3 | 43,369,470 | 6,505,420,500 | 43,343,404 | 6,204,588,766 | 98.33% | 95.29% | 45.04% |
| WT-1 | 43,350,434 | 6,502,565,100 | 43,302,216 | 6,214,572,200 | 98.45% | 95.59% | 44.77% |
| WT-2 | 43,363,820 | 6,504,573,000 | 43,336,418 | 6,280,836,666 | 98.23% | 95.12% | 44.90% |
| WT-3 | 43,343,924 | 6,501,588,600 | 43,317,556 | 6,252,894,432 | 98.23% | 95.09% | 44.90% |

**Supplementary Table 2. Primers used for RT-qPCR**

| Gene | Forward (5'->3') | Reverse (5'->3') |
| --- | --- | --- |
| *Actinidia06502* | GGTCCTTGGCTTGTTTCAAAGG | GCCTCCCAGAAGATTCAGCA |
| *Actinidia28004* | AAGTGGTCTGGCGTAACTCG | AGTGACAAACCACCAACCGT |
| *Actinidia02117* | GTCCTCGCCAAGGGGTTTTA | TCCCAATACCCCACTCCGAC |
| *Actinidia34575* | ATGCACTGAAGTTGTTTGGTGG | CCTTGGCATGCTTTTGGGTG |
| *Actinidia26771* | TCCGGGCTGGCACAG | GTGGACACGCGAATCACG |

**Supplementary Table 3. The extent of boron transport from leaf to root and stem to root in kiwifruit seedlings under varying boron levels.** Different letters indicate significant differences (Duncan’s multiple range test, *P* < 0.05).

| ***AC***  TFs leaf/root | WT | 0.6 mg/L B | | 3mg/L B | 6mg/L B | 9mg/L B |
| --- | --- | --- | --- | --- | --- | --- |
|  | 3.69±0.32^g^ | 2.1±0.05^e^ | 1.75±0.61^cd^ | | 2.55±0.037^f^ | 2.24±0.042^ef^ |
| TFs stem/root | 1.96±0.14^ef^ | 1.08±0.033^a^ | 1.25±0.047^ab^ | | 1.57±0.03^bc^ | 1.36±0.027^ab^ |
| ***AA*** |  |  |  | |  |  |
| TFs leaf/root | 2.87±0.29^bc^ | 3.94±0.08^c^ | 3.93±1.42^c^ | | 2.82±0.13^bc^ | 2.63±0.04^abc^ |
| TFs stem/root | 1.4±0.12^ab^ | 1±0.083^a^ | 2.4±0.73^abc^ | | 1.23±0.019^ab^ | 1.27±0.03^ab^ |


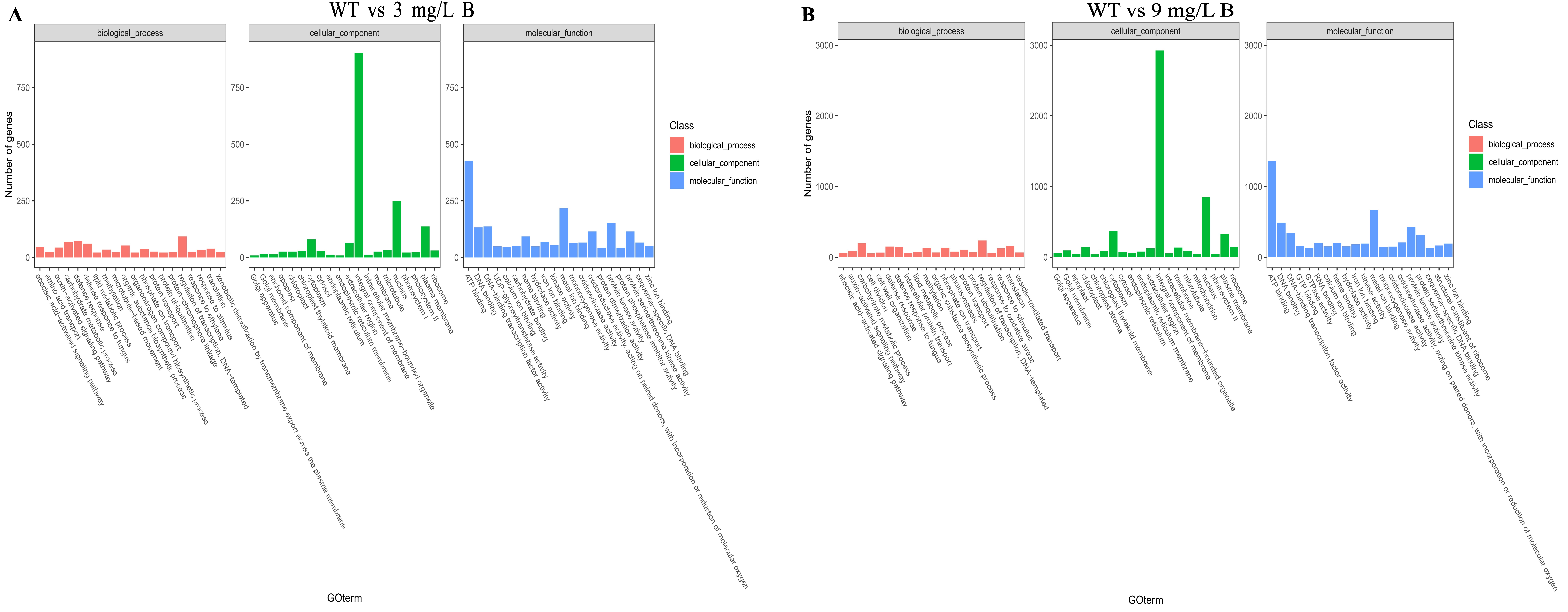


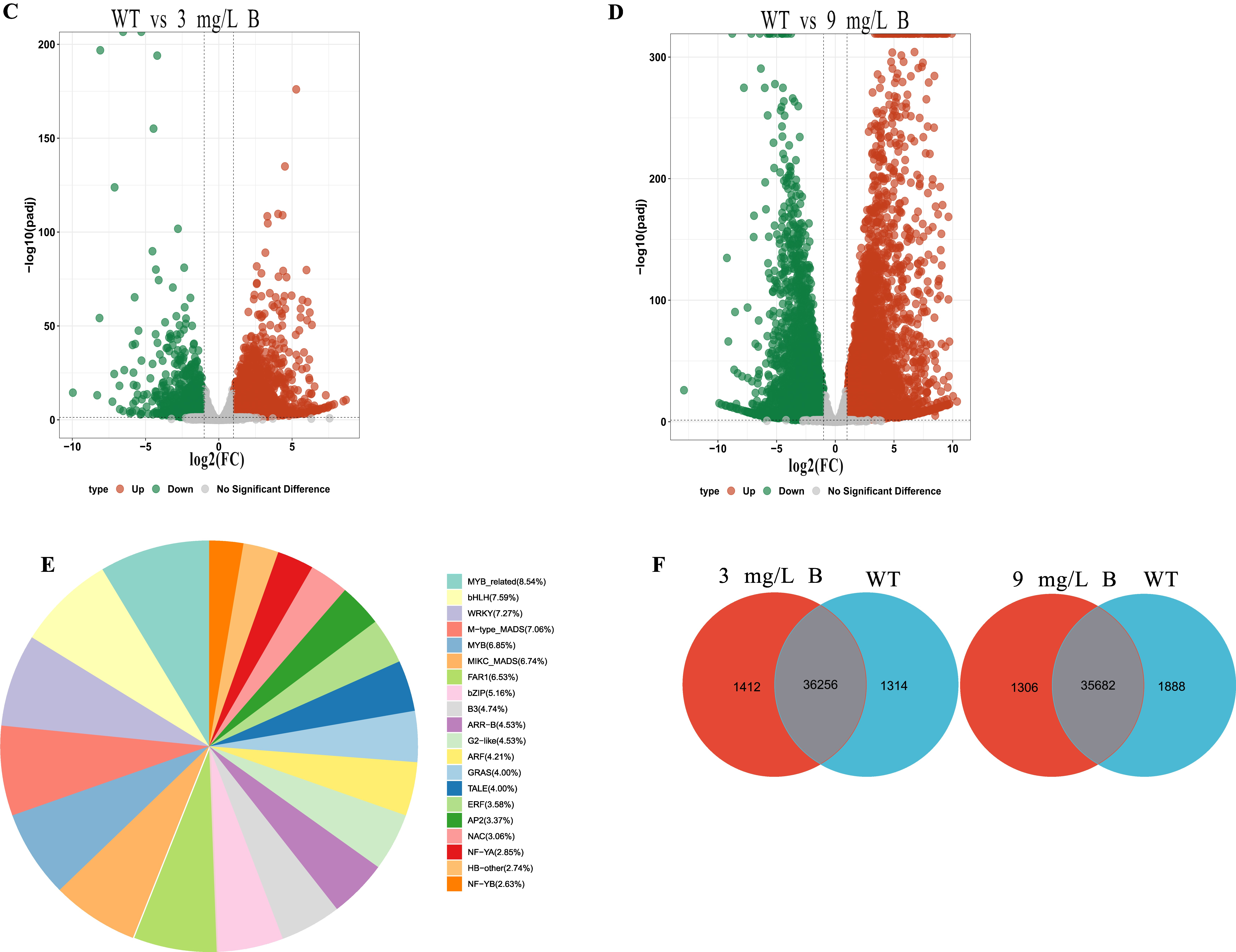


**Supplementary Figure 1. Differentially expressed genes (DEGs) of *Actinidia arguta* related to B stress.** A) GO terms: WT vs 3 mg/L B and B) WT vs 9 mg/L B, C) volcano plots of DEGs WT vs 3 mg/L B and D) WT vs 9 mg/L B, E) TFs, F) Venn diagram of DEGs of WT vs 3 mg/L B and WT vs 9 mg/L B.


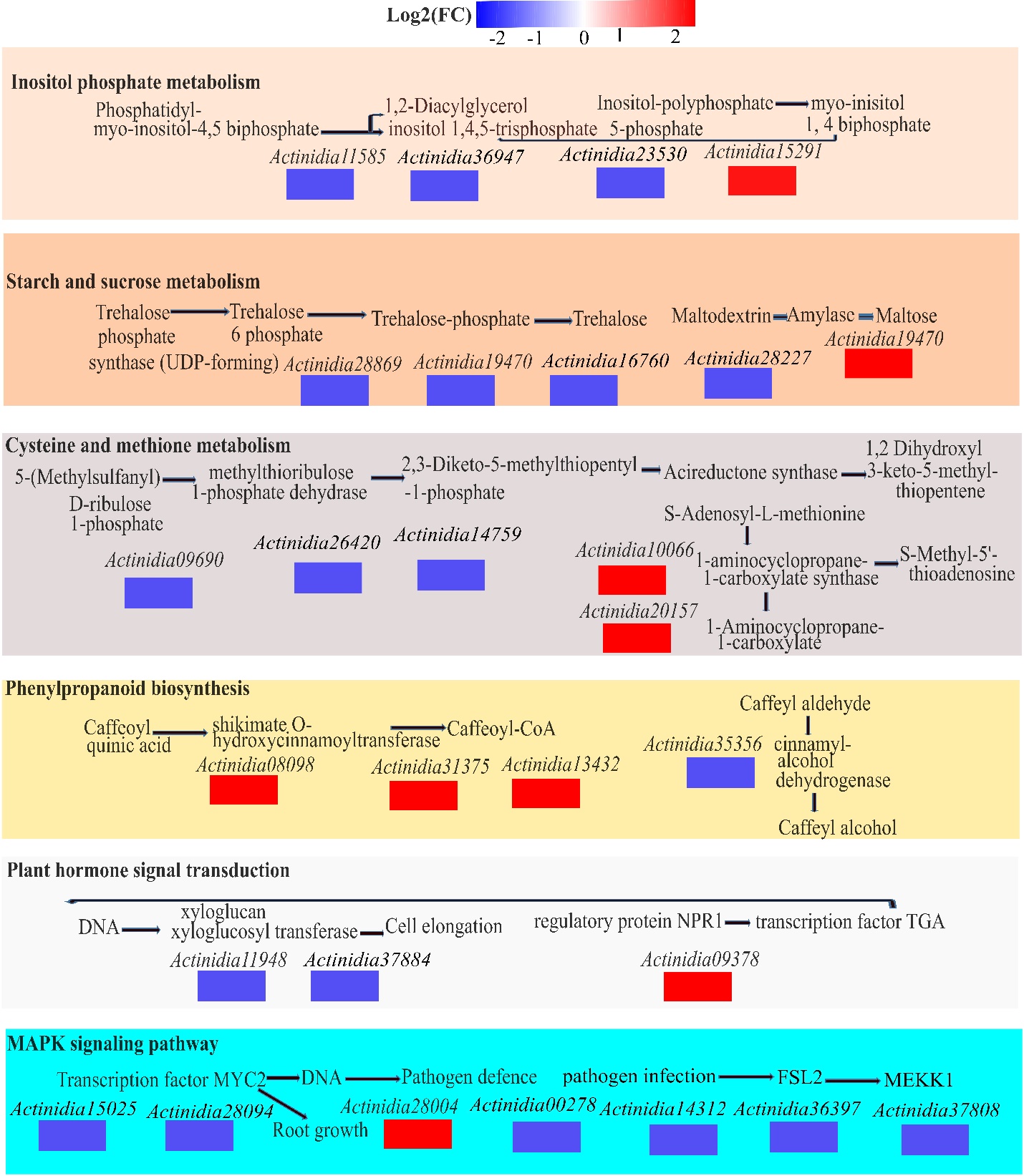


**Supplementary Figure 2. The specific pathways of kiwifruit (*Actinidia*) genes involved in boron responses.** A red cell denotes an upstream fold change and a blue cell indicates a downstream fold change in gene expression.


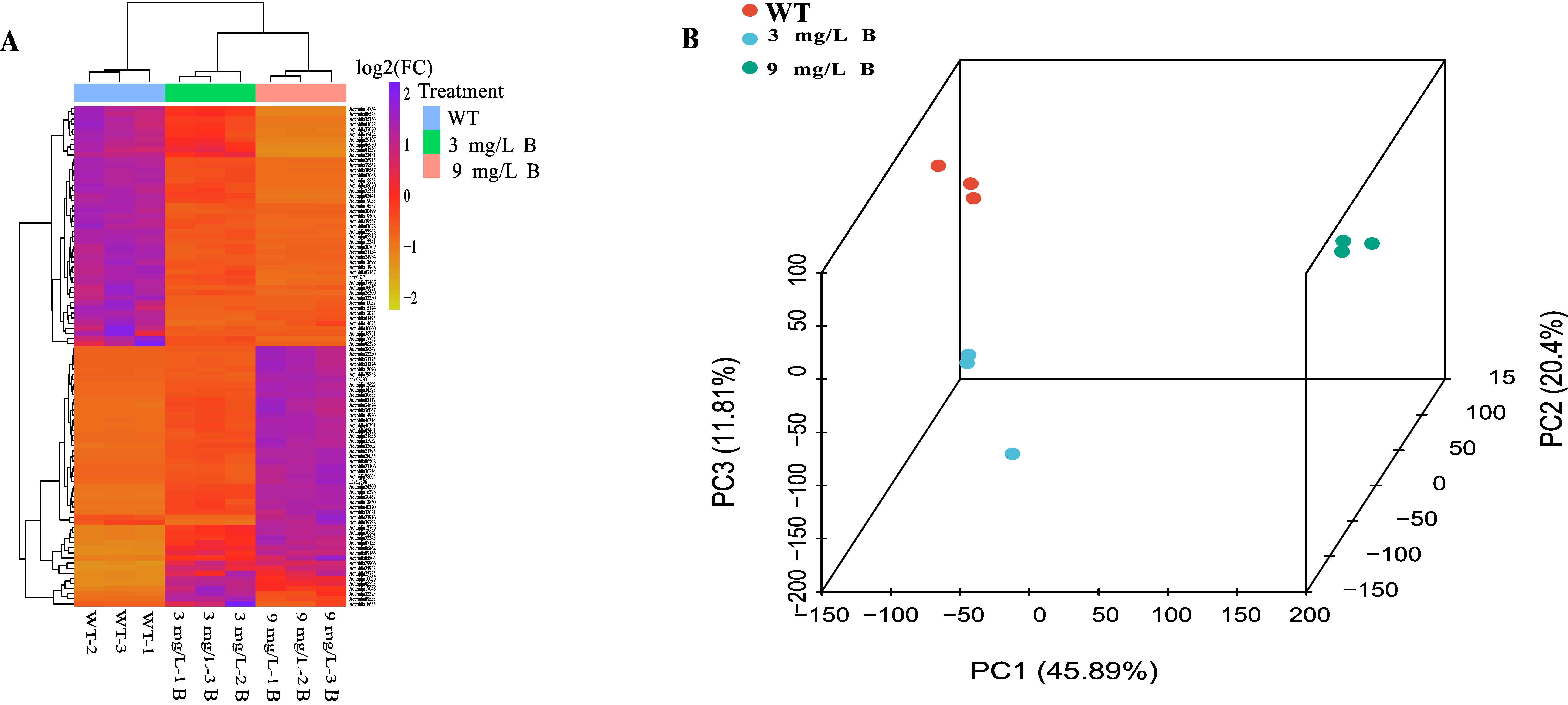


**Supplementary Figure 3.**  **A) Heatmap of DEGs, with purple for upregulated, red for medium, and yellow for downregulated genes, B) Principal component analysis (PCA).**

**
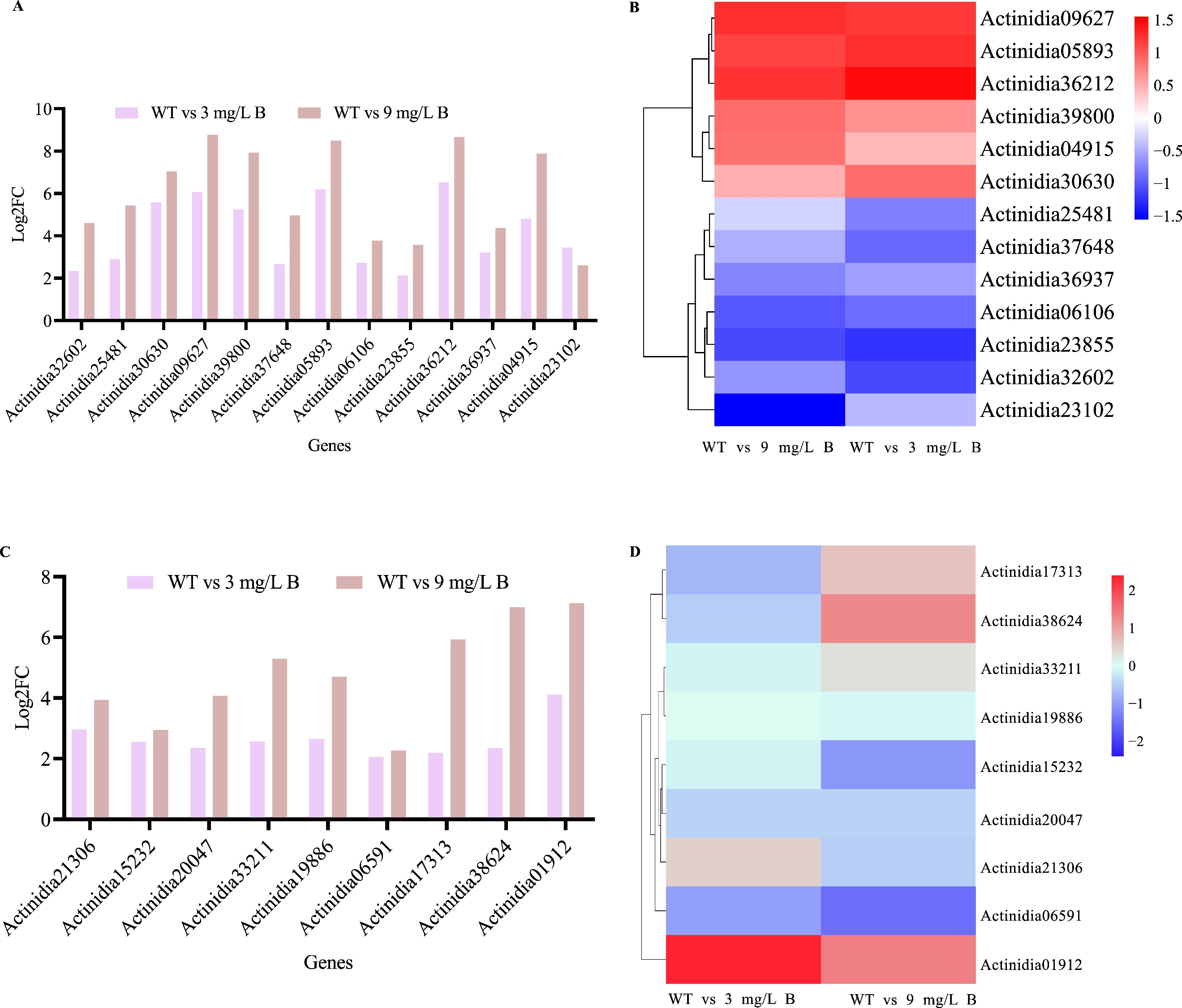
**

**Supplementary Figure 4.** **The differentially expressed genes (DEGs) categorized as transcription factors encompass the following:** A) Bar plot representing the WRKY gene family, B) Clustered heatmap analysis of the WRKY gene family, C) Bar plot illustrating the NAC gene families, D) Clustered heatmap analysis of the NAC gene families in kiwifruit subjected to varying levels of boron. Within the heatmap, red signifies higher concentrations of genes, while blue denotes lower concentrations.
